# Oriented to Multi-Branched Structure Unsupported 3D Printing Method Research

**DOI:** 10.3390/ma13092023

**Published:** 2020-04-26

**Authors:** Qingxi Hu, Die Feng, Haiguang Zhang, Yuan Yao, Mohamed Aburaia, Herfried Lammer

**Affiliations:** 1 Rapid Manufacturing Engineering Center, Mechatronic Engineering and Automation of Shanghai University, Shanghai 200444, China; 2 Shanghai Key Laboratory of Intelligent Manufacturing and Robotics, Shanghai University, Shanghai 200072, China; 3 Competence Center Digital Manufacturing and Robotics, University of Applied Science Technikum Wien, Höchstädtplatz 6, 1200 Wien, Austria; 4 Kompetenzztentrum Holz Gmbh, Altenberger Straße 69, 4040 Linz, Austria

**Keywords:** non-directional unsupported 3D printing, multi-branched structure, five-axis, model decomposition

## Abstract

For the traditional three-axis (3D) configuration of the additive manufacturing (AM) platform, when printing the target model with a multi-branched structure, it is imperative to construct adequate support structures. To eliminate the use of support during the printing process, a non-directional unsupported 3D printing method for five-axis AM is proposed in this paper. By carrying out the K-means clustering algorithm, the coarse partition of the model is obtained, and then the fine decomposition represented by a sequence of separating planes is determined by a local dynamic search adjustment algorithm according to manufacturing constraints. The multi-branched structure of the model is divided into simple subparts so that the general model can be built in different directions and be printed with its own parts as the support. Two case studies were carried out for verification. The experimental results showed that the branch-model can be printed without support using the non-directional unsupported 3D printing method, and the non-directional unsupported 3D printing can save 18.72–20.60% of materials and 20.60–23.33% of time compared to conventional 3D printing.

## 1. Introduction

With its rapid development over the last 30 years, 3D printing technology that fabricates objects through the deposition of a material using a print head, nozzle, or another printer technology [1] is widely used in a variety of applications ranging from the micro-scale fabrication of biochemical structures to the large-scale construction of architecture [2]. 3D printing devices, especially the ones based on fused deposition modeling (FDM) (which is a material extrusion (ME) process [1]), are now available for the consumer-level market. Though it claims the ability to fabricate any shape with high complexity, some models containing suspension, hollow, or other multiple branches often need additional support structures to ensure smooth printing [3]. In prior research, the drawbacks of additional supports during fabrication have been extensively studied: Briefly, the addition of support structures mainly causes material waste, a slower speed of printing, and surface damage [4,5,6,7].

To overcome the limitation of traditional additive manufacturing (AM), more and more researchers have been exploring both software and hardware solutions to eliminate the usage of supporting structures. For the former, Ke Xu [8] proposed a novel multidirectional process planning algorithm for five-axis AM; the core of the strategy was to decompose the model into support-free parts directly pertaining to the cusp-height constraint, each with its own build direction. Jun Zhang [9] developed an adaptive slicing algorithm that could export optimal slices without support structures for five-axis hybrid layered manufacturing. Jun Zhang’s algorithm calculated the normal vector of the random points by selecting a series of random points on the cantilever surface, and then a Gaussian mapping method was used to find the optimal construction direction. Ding et al. [10] proposed a decomposition–regrouping slicing method for parts with a large number of holes. The method first used the simple curvature-based volume decomposition to decompose the model into sub-volumes, and then it focused on regrouping these sub-volumes. Muntoni et al. [11] recently proposed a decomposition algorithm for processing general 3D geometries into a small set of non-overlapped height field blocks, where the directions of height-fields were constrained to the major axes to solve the overlap problem; as a result, the generated height-field blocks could be fabricated by molding or 3D printing. The use of a model auto-partitioning algorithm for generating three-dimensional layer information to build overhang/undercut features was proposed by Lee et al. [12]. The cantilever portion divided the original model, followed by the slicing of the divided sub-models separately. Ding et al. [13] developed a path planning methodology that aimed to generate tool-paths for an eight-axis robotized laser-based direct metal deposition system. Taking advantage of the additional two-axis tilt and rotatory system, the tool-paths of overhanging structures of a revolved part were mapped at a planar base. However, it highly depended on the eight-axis hardware system. Dai et al. [14] presented a new method to fabricate 3D models on a robotic printing system equipped with multi-axis motion. Materials were accumulated inside the volume along curved tool-paths so that the need for supporting structures could be tremendously reduced. However, the surface of the printed model was very rough.

On the other hand, researchers have spent great effort to renovate the hardware for more capabilities. In a recent work called RevoMaker [15], a standard low cost FDM printer was modified and combined with a rotating cubic shape platform, and this finally achieved multi-directional 3D printing. However, the cuboidal platform used in their system could not print general freeform models. Lee W et al. [16] designed a five-axis printing platform based on 3D printers. He modified the Arduino firmware of the original 3D printing platform, added two additional rotating shafts (axes *A* and *C*), and used Java language to write the custom five-axis G code. However, due to the extra weight of mounting the *C* axis, the platform still had some problems related to the precision of the rotation axis. Pan et al. [17] proposed a five-axis motion system similar to five-axis CNC (Computerized Numerical Control Machining Center) machining to accumulate materials. A six-DOF (Degrees Of Freedom) parallel kinematic Stewart platform was presented in the work of Song et al. [18] for multi-directional AM. Only small components with simple shapes were fabricated in these two systems.

In summary, research to date has mainly focused on developing new multi-axis slicing algorithms and adding more degrees of freedom into motion, whereas algorithms aimed at multi-axis printing system required a large amount of calculations and were hard to apply to actual printing. In this paper, the main aim was to present a non-directional unsupported 3D printing method oriented to a multi-branched structure; in this process, the target model is segmented into different regions based on K-means clustering algorithm, according to the pre-decomposition results; the fine decomposition is carried out by a limited local dynamic adjustment algorithm, and then each subpart of model can be sliced along a direction in a support-free way. With the help of the model decomposition, the method can use its own materials as the support so as to avoid the external and internal support structure of the overhanging branch, reduce the printing consumables and time, avoid the post-processing process of stripping the support, and, finally, improve the flexibility of the unsupported printing process.

## 2. Materials and Methods

### 2.1. Overview of Non-Directional Unsupported 3D Printing Strategy

**Figure 1:**
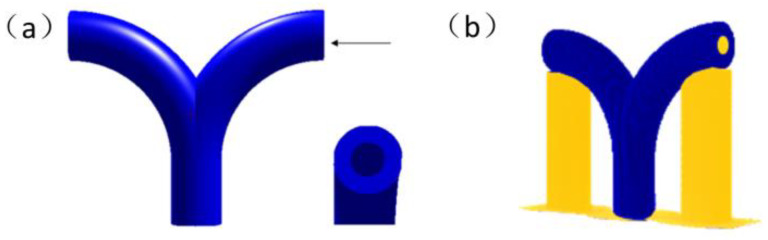
(**a**) The two-branches pipe model. (**b**) Support for 3D slicing process of the two-branched pipe model.

The above problems were mainly applied to unsupported 3D printing with the multi-branched structure. As shown in Figure 1a, a two-branched pipe model with regular circular through holes is shown.

The multi-branched structure model would inevitably produce support structures when sliced in ordinary 3D. As shown in Figure 1b, the model was imported into the Repetier-Host software and sliced by the conventional 3D printing Cura slice engine. The blue part is the targeted printing model part, and the yellow part is the printing support part. It can be found in the figure that there are nearly twice the materials consumed in the printing process by the ordinary slicing way than the model body, and the supporting materials in the through-hole of the model are difficult to peel off. Even if the supporting materials can be peeled off, the peeling device can easily scratch the surface of the printed piece, and the peeling process requires a lot of physical effort and time. At the same time, the branch part is a hollow bend with a certain curvature that can hardly avoid support in the traditional 3D printing method.

**Figure 2:**
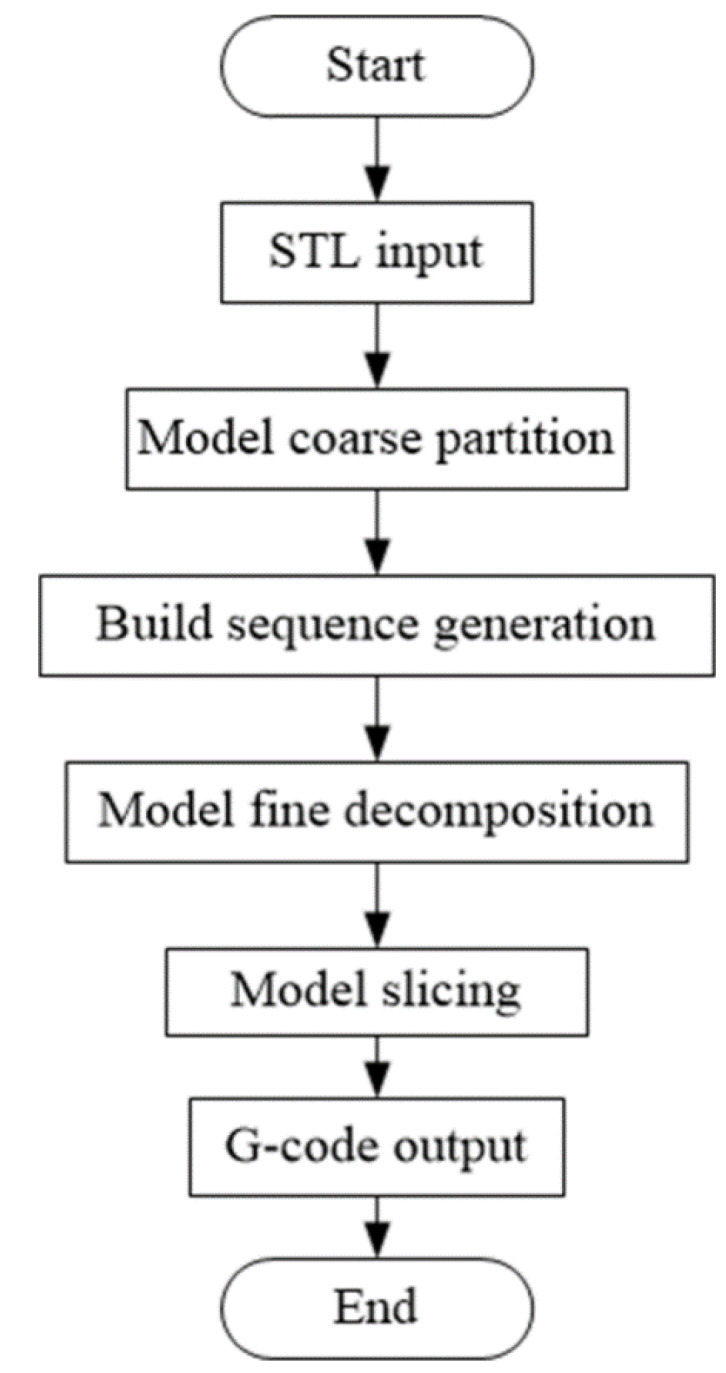
Flow chart of the non-directional unsupported 3D printing method.

On the contrary, the non-directional unsupported 3D printing method based on model decomposition toward five-axis AM can use printing materials as the base materials to realize self-support by partitioning the model, slicing the subpart, adding two degrees of freedom to the receiving platform of the printer, and utilizing the spatial movement of the platform. According to the input three-dimensional model, the model is preliminarily partitioned by K-means clustering. Based on the results of coarse partitioning and building sequence, the fine decomposition of the original model is carried out by a local dynamic search adjustment method considering manufacturing constraints, each subpart of the model can be sliced along a direction in a support-free way, and the spatial printing G code of each part is obtained. Finally, the G code is combined with the association information between subparts. The specific process of the non-directional unsupported 3D printing method is shown in Figure 2:

#### 2.1.1. STL Input

STL (Standard Triangle Language) is the file format for model data describing the surface geometry of an object as a tessellation of triangles [1]. The commercial software Solidworks is used to design the multi-branched structure model and to export the STL file. Two formats can be used for STL files: the ASCII (American Standard Code for Information Interchange) format or binary [19]. Then, a program in a Python script is written to read the triangular slices in the STL file; this is saved in the computer’s cache with the data list.

#### 2.1.2. Model Coarse Partition

To perform model partition, the input point list of the model is classified into different regions using the K-means clustering algorithm [20], which is a kind of iterative clustering analysis algorithm. The algorithm randomly selects K objects of the data as the initial clustering center. Then calculates the distance between each object and each clustering center, and assigns each object to the nearest clustering center. Finally the data are divided into K regions. This step outputs approximate meaningful partition from the input model.

#### 2.1.3. Build Sequence Generation

As the model is preliminarily divided into coarse subparts, the printing sequence of subpart is determined based on the breadth first search (BFS) algorithm, which is one of the simplest graph search algorithms [21]. The relationship between the subparts can be displayed in the undirected graph, and the undirected graph can be turned into a directed graph by using the BFS algorithm. The direction on the graph represents the printing sequence. This step can ensure subsequent collision-free printing.

#### 2.1.4. Fine Decomposition

Though the coarse partition separates the model into different regions, the boundaries between subparts are not clear and flat, and AM is based on layers. To get the definite plane that separates the model into subparts, a local dynamic search adjustment method that considers the manufacturing constraints is carried out to implement further decomposition; the final separating plane must satisfy the printing condition that makes collision-free and support-free printing on the five-axis AM system feasible.

#### 2.1.5. Model Slicing

Each subpart is sliced by a five-axis dynamic slicing algorithm [22]; by extracting the skeleton curve of the subpart, the equations of the skeleton curves are achieved, and these are used for the construction curve. Then, the model is sliced by a series of dynamic planes calculated by the construction curve.

#### 2.1.6. G-Code Output

As the spatial printing G code of each part is obtained by model slicing, the integral G-code is combined with the association information between subparts.

### 2.2. Non-Directional Unsupported 3D Printing Method

#### 2.2.1. Model Coarse Partition

Model decomposition is a technique of geometry processing. This paper achieved model coarse partition based on the K-means clustering algorithm, which is a typical distance-based clustering algorithm. The K-means clustering partition of a three-dimensional point cloud model can avoid the generation of a large number of relatively small segmented pieces; that is, no excessive number of segments can be generated, and at the same time, approximate meaningful partition results can be obtained.

Three-dimensional models are stored as STL files, which represent the three-dimensional model in the form of triangular faces. Then, the STL model file is extracted as a point cloud model file. The center of the gravity position vector of triangular faces is extracted and marked as {$x_{1}$,${\;x}_{2}$,…,${\;x}_{n}$}, where $\varphi_{i}^{m}$ represents the class i of the m-th merger. The final number of clusters K is determined beforehand. The specific K-means clustering process is as follows:

Input: Clustering numbers K, the gravity position vector of triangular faces: {$x_{1}$,${\;x}_{2}$, …,${\;x}_{N}$}.

Output: Clustering partition: $\varphi =\varphi_{1}\cup \varphi_{2}\ldots \cup \varphi_{k}$, where $\varphi_{i\;},\left(i=1,2\ldots ,k\right)$ represents a sub-region of the model.

Step 1 K feature vectors are selected from the set of the center of the gravity position vector of triangular faces, marked in $z_{1}^{0}$,${\;z}_{2}^{0}$, …, $z_{k}^{0}$ as the initial clustering center.
Step 2 According to the principle of minimum distance, each feature vector is divided into K classes. The distance between the center of the feature vector $x_{i}$ and $Z_{j}^{m}$, which is the center of the class $\varphi_{j}^{m}$, is marked as $d_{\mathrm{il}}^{m}$, if the distance satisfies the following equation:
  (1) $$d_{\mathrm{il}}^{m}=\underset{j}{\min}\left[d_{\mathrm{ij}}^{m}\right],\;i=1,2\ldots n$$
Step 3 As a result, $x_{i}$ is considered to belong to $\varphi_{l}^{m+1}$ in the new clustering results. Note that $\underset{j}{\min}\left[d_{\mathrm{ij}}^{m}\right]$ represents the minimum distance during the distances between the center of the feature vector $x_{i}$ and $Z_{j}^{m}$.
Step 4 New clustering centers are recalculated; these are the average of all feature vectors in the new clustering results.
Step 5 Repeat Steps 2–3 until the new class center is the same as the previous one or the number of clusters is larger than the preset K value;otherwise, the clustering iteration ends.

**Figure 3:**
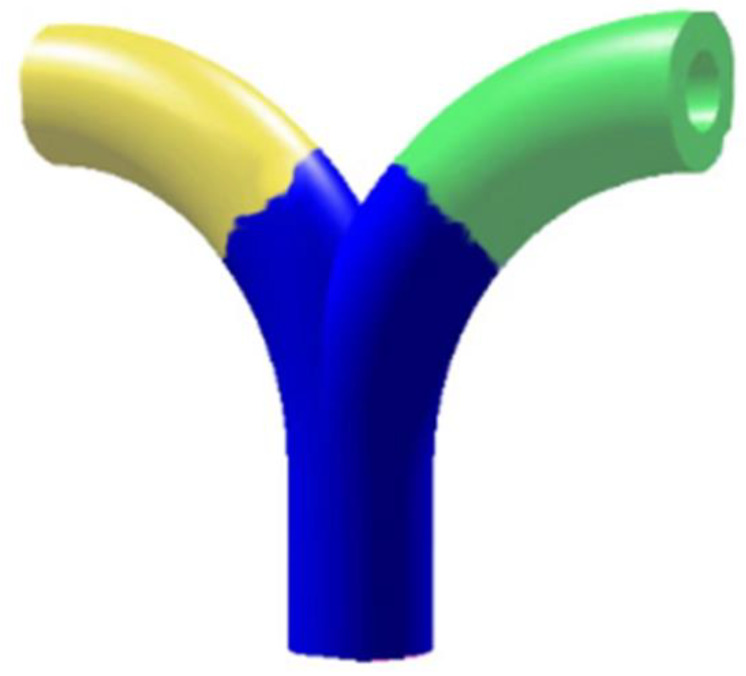
K-means clustering meaningful partition of the model.

As shown in Figure 3, the result of K-means clustering partition result is ideal, as an approximately meaningful partition is obtained and the original model is decomposed into three regions; the branch parts are basically separated from the main body of the model. However, it could be found that the boundaries between three regions are jagged and uncertain. As the 3D printing technology accumulates materials layer by layer, the fine decomposition is imperative to achieve a flat separating plane.

#### 2.2.2. Determining the Model Printing Sequence

Intuitively, the printable features are identified individually and sequentially from the top down, while the building sequence is in the reverse order, such that the formerly printed subpart served as the base for the later ones. The result of a coarse partition can be converted into an undirected graph according to the neighboring information between parts. The BFS sequence planning strategy perfectly suits parts with a linear feature hierarchy. BFS can be defined as follows: starting from a selected node v, then visiting all the adjacencies of node v until all vertices in the graph that have paths to the source node v have been accessed. At this point, the search process starting with node v is over.

**Figure 4:**
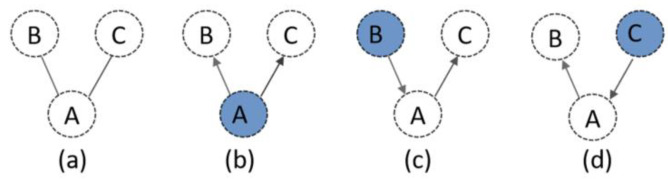
Printing sequence planning graph: (**a**) an undirected graph as the result of coarse decomposition, (**b**) a directed graph if the sequence start from node ‘A’, (**c**) a directed graph if the sequence start from node ‘B’, and (**d**) a directed graph if the sequence start from node ‘C’.

Mark model as φ; the coarse partition decomposes the model into n parts, which can be expressed as: $\varphi =\varphi_{1}\cup \varphi_{2}\ldots \cup \varphi_{n}$. Specifically, each part $\varphi_{i\;}\;\left(i=1,2\ldots ,n\right)$ is considered as a node in a graph. When $\varphi_{i}$ and $\varphi_{j}$ are connected, an undirected edge is constructed in a graph to represent this relationship. For example, in the model is shown in Figure 4, its undirected graph marked G can be defined as what is shown in Figure 4a. Starting from a selected node (e.g., node ‘A’ in Figure 4b), a sequence of nodes on the graph can be generated by adopting the BFS algorithm. The order of visit generates the directions of edges, and G is converted into a directed graph $\vec{G}$ with the starting node named as a root. In thecurrent implementation, the root is interactively selected by users. When starting from a different node, a different graph can be generated (e.g., Figure 4c,d).

#### 2.2.3. Fine Decomposition

(1) Definition of Fine Decomposition Problem

After getting the sequence of printing based on the coarse decomposition, when two adjacent parts $\varphi_{k}^{+}$ and $\varphi_{k}^{-}$ are separated by a plane $\tau_{k}$ and have the printing sequence of $\varphi_{k}^{-}$, followed by $\varphi_{k}^{+}$, the oriented plane $\tau_{k}$ is named as the base plane for the fabrication of $\varphi_{k}^{+}$, and the cross-section $\tau_{k}$ ∩ φ is called the base cross-section ${\;S}_{i}$. In this case, the fabrication of the part $\varphi_{k}^{+}$ starts to accumulate materials on the base cross-section ${\;S}_{i}$.

**Figure 5:**
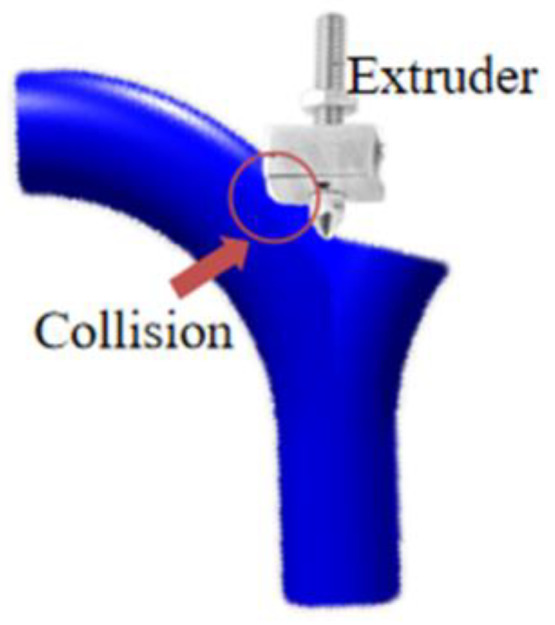
Collision between the nozzle and the printed model occurs.

For parts with multiple branches, this exact reverse building order may encounter the interference issue between the nozzle head and the already printed part. As illustrated in Figure 5, the nozzle collides with the printed part to avoid collision while printing, and the cross-section formed by the separating plane should not intersect with the already printed part. On the other hand, the base cross-sections play the role of accumulating the subpart $\varphi_{k}^{+}$, and all base cross-sections need to face up. As a result, two constraints to prevent collision during printing are:

- Constraint I: The base cross-sections formed by the separating planes cannot intersect with printed part of model.
- Constraint II: All base cross-sections need to face up.

(2) A Local Dynamic Search Adjustment Method

**Figure 6:**
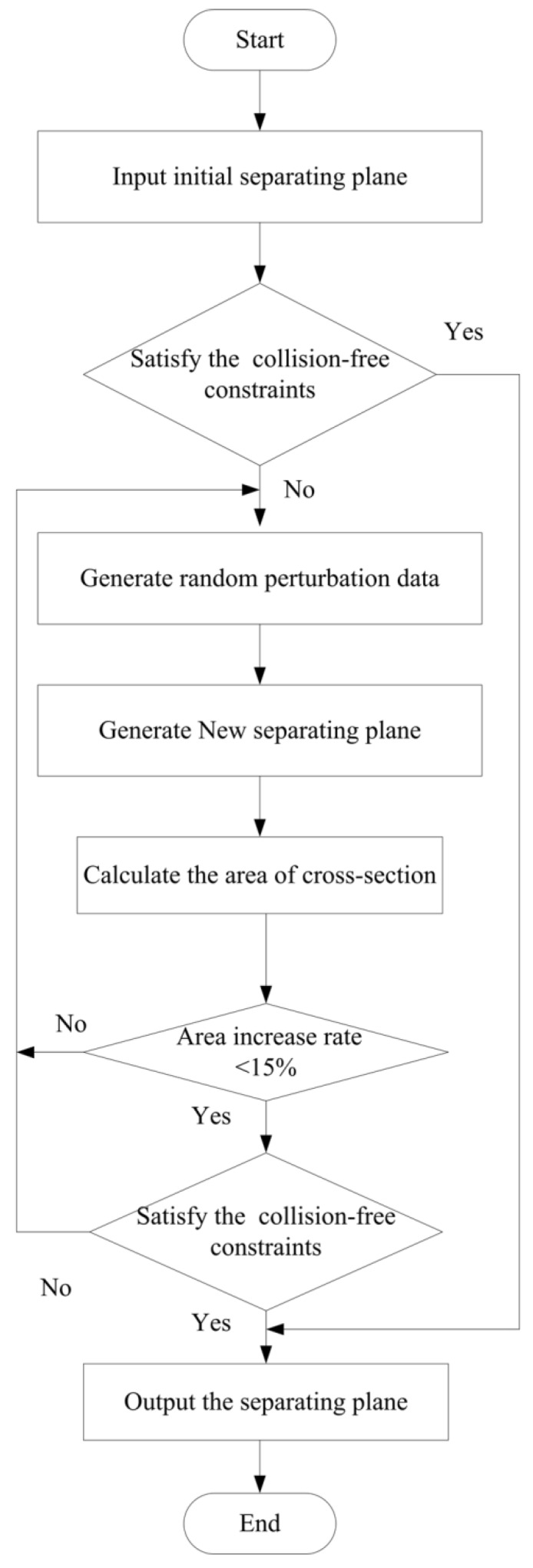
Process to determine the practicable separating plane.

A local dynamic search adjustment method is carried out to implement fine decomposition, the process of which is shown in Figure 6.

The specific steps execute are as follows:

Step 1: Sample the boundary curve between two segmented patches into three points that are not on the same line and which are selected randomly from the previous sampling points; three points can define a plane. The initial separating plane $\tau_{k}$ is expressed as:
  (2) $$A_{k}x+B_{k}y+C_{k}y+D_{k}=0$$
  The normal vector of the initial separating plane is marked as $n_{k}=\left(r,\varphi ,\theta \right)$.
Step 2: Check the initial separating plane with collision-free constraints. If the constraints are satisfied, output the initial separating plane. Otherwise, continue Step 3.
Step 3: Small random perturbation is added to the initial separating plane; the perturbed data include the offset distance $d_{o}$ and the normal vector $n_{k}$ of the plane. The new separating plane is generated along the normal vector of the plane with a very small offset value, and the offset value is randomly generated between 0 and 2 mm. The offset direction is also randomly determined. It has two choices, one along the positive direction of the normal vector and the other along the negative direction. At the same time, the normal vector can be expressed as (r,φ,θ) in space polar coordinates by changing the angle of φ and θ within a 5° angular variation in the randomly used counterclockwise or clockwise directions, and the direction of the normal vector is controlled, thus generating new separating planes.
Step 4: Calculate area of new base cross-section formed by the perturbed parameters. If the perturbed new parameters lead to a more than 15% area increase in the cross-section that is excluded, the new perturbed parameters should be abandoned, then return to Step 3; otherwise, continue Step 5.
Step 5: Check the new separating plane with collision-free constraints. If the constraints are satisfied, output the new separating plane. Otherwise, return to Step 3.

**Figure 7:**
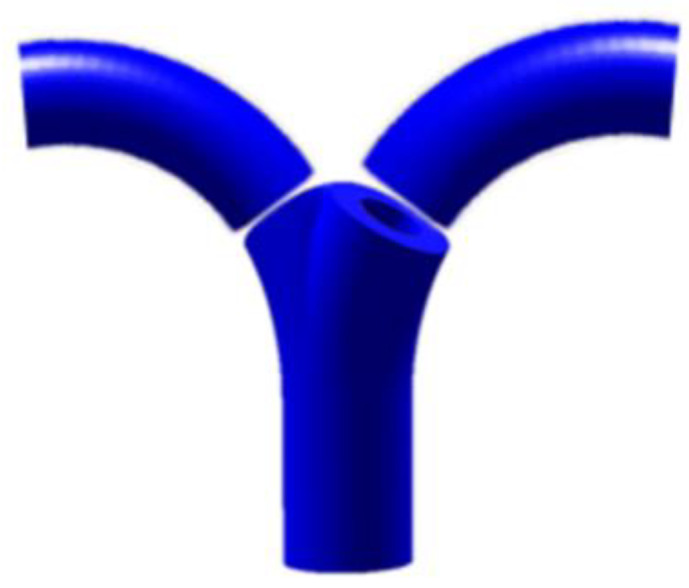
Fine decomposition result of the model.

Finally, a cutting plane approximating the boundary between two patches is obtained. These planes now form a fine decomposition of the input model φ. The final result of the decomposition is shown in Figure 7.

#### 2.2.4. Model Slicing

After the multi-branched model is divided into different parts, each subpart should be sliced to obtain the printing G-code. Considering the shape characteristics of some parts of the model are complex, this paper adopted a five-axis dynamic slice algorithm [19]. By extracting the skeleton curve of the model, the equation of the skeleton curve can be acquired, and the tangent vector of each slicing point on the skeleton cure is taken as the slice direction. The specific implementation steps are as follows:

(1) Achieve the Equation of the Skeleton Curve

The skeleton point of the model is extracted using the mean curvature flow [23] algorithm, and then the extracted skeleton point set is fitted with a space curve to obtain a skeleton curve of the model. This paper fits the neutral skeleton point set using the least square method [24]. Assume that skeleton point set positions are given as p_k_ (x_k_, y_k_, z_k_), where k can take any integer value from 0 to n.

Firstly, figure out the equation of input variables $\left(x_{i},y_{i}\right),\;i=0,1\ldots n$. The least square method figures out the only function $s^{*}\left(x\right)$ that satisfies the following equation:

(3) $${\left\|\delta \right\|}_{2}^{2}=\overset{m}{\underset{i=0}{\sum }}\delta^{2}=\overset{m}{\underset{i=0}{\sum }}w\left(x_{i}\right){\left[S\left(x_{i}\right)-y_{i}\right]}^{2}$$

${\left\|\delta \right\|}_{2}^{2}$ values should be the minimum. In the function, $w\left(x_{i}\right)$ represents the number of repeated observations at point $\left(x_{i},y_{i}\right)$. In the same way, calculate the equation of points $\left(x_{i},z_{i}\right)\;i=0,1\ldots ,n$. The equation of the space curve can be written as follows:

(4) $$\{\begin{array}{c} y=\overset{n}{\underset{k=0}{\sum }}a_{k}\cdot x^{k}\\ z=\overset{n}{\underset{k=0}{\sum }}b_{k}\cdot x^{k} \end{array}$$

The above general equations can be converted into parametric equations of space curves p(u):

(5) $$\{\begin{array}{c} x=x\left(u\right)\\ y=y\left(u\right)\\ z=z\left(u\right) \end{array}\;u\in \left[0,1\right]$$

(2) Calculate the Slicing Parameters

Every slicing point is determined by the length of the skeleton curve that can approximately represent the five-axis composite printed stack thickness scalar and the provided layer thickness t. The length of the curve can be expressed as follows:

(6) $$s\left(u\right)=\int_{0}^{u}\sqrt{x^{\prime }{\left(u\right)}^{2}+y^{\prime }{\left(u\right)}^{2}+z^{\prime }{\left(u\right)}^{2}}\;\mathrm{du}=i\cdot t=s$$

where i is the slice layer number and t is the slice layer thickness.

Next, find the slicing point of the slice and the tangent of the point location. According to Equation (4), the current point relative parameters  u can be calculated as $u_{c}=f\left(i,t\right)$; therefore, the coordinates of the intersection point of current slice and skeleton curve are $p_{c}\left(x\left(u_{c}\right),\;y\left(u_{c}\right),\;z\left(u_{c}\right)\right)$, and the intersection tangent vector at this point can be expressed as $\tau_{c}\left(x^{\prime }\left(u_{c}\right),y^{\prime }\left(u_{c}\right),z^{\prime }\left(u_{c}\right)\right)$.

The coordinates of the intersection point of each slice and the intersection tangent vector data of the fitted skeleton curve are calculated, and these data are constructed as a slice parameter object.

(3) Dynamic Model Slicing

**Figure 8:**
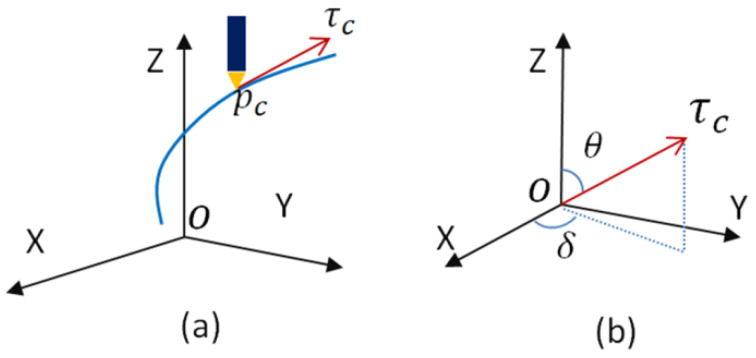
Diagram of the magnitude of the *A*- and *C*-axes: (**a**) current printing point; (**b**) the formation of the rotation angle.

According to the machine equipment used in this experiment, the nozzle of the machine is always in the vertical direction. In order to keep the direction of the nozzle along the direction of the point of the vector, the rotation angle of the platform’s *A* and *C* axes are needed to be calculated. The dataset of the model is accompanied by the rotation of the platform. As shown in Figure 8, the diagram illustrates the positional relationship between the nozzle head and the printed dot direction vector; assuming the traversal point is $p_{c}\left(x\left(u_{c}\right),y\left(u_{c}\right),z\left(u_{c}\right)\right)$, the tangent vector $\tau_{c}\left(x^{\prime }\left(u_{c}\right),y^{\prime }\left(u_{c}\right),z^{\prime }\left(u_{c}\right)\right)$ at the point $p_{c}$ is used to determine the rotation angles of the five-axis printing *A*- and *C*-axes. The rotation angles of the *A*-axis are:

(7) $$\theta =\arccos\frac{z^{\prime }\left(u_{c}\right)}{\left|P{\left(u\right)}^{\prime }\right|}$$

and the rotation angles of the *C*-axis are:

(8) $$\delta =\arctan\frac{y^{\prime }\left(u_{c}\right)}{x^{\prime }\left(u_{c}\right)}$$

When the *A*- and *C*-axes are rotated, the spatial position of the model changes, so the spatial position transformation matrix is used to transform the triangular list data and the neutral curve of the model in real-time. In every slice, rotate the model along with the intersection point of the *A*- and *C*-axes; the rotation matrix around axes *A* and *C* is:

(9) $$\mathrm{Rot}=\mathrm{Rot}\left(A\right)\cdot \mathrm{Rot}\left(C\right)=\left[\begin{array}{cccc} 1 & 0 & 0 & 0\\ 0 & \cos\theta & -\sin\theta & 0\\ 0 & \sin\theta & \cos\theta & 0\\ 0 & 0 & 0 & 1 \end{array}\right]\cdot \left[\begin{array}{cccc} \cos\delta & -\sin\delta & 0 & 0\\ \sin\delta & \cos\delta & 0 & 0\\ 0 & 0 & 1 & 0\\ 0 & 0 & 0 & 1 \end{array}\right]$$

**Figure 9:**
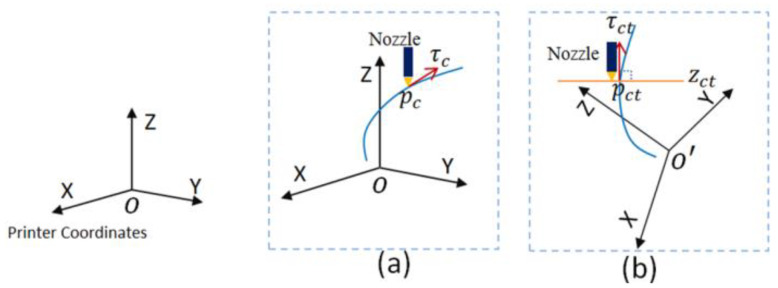
Schematic diagram of model space rotation: (**a**) transform before and (**b**) transform after.

The diagram in Figure 9, shows the schematic before and after model transformation. After each slice, the model needs to be updated by matrix transformation:

(10) $$P_{\mathrm{CT}}=\mathrm{Rot}\cdot P_{C}$$

where $P_{C}$ represents the data before the transformation and $P_{\mathrm{CT}}$ represents the data after the transformation.

When the rotation of the platform satisfies that the direction of the slicing point is in the vertical direction (i.e., the direction of the nozzle), the rotated STL structure is sliced along the Z coordinate plane of the transformed key point $P_{\mathrm{CT}}$.

#### 2.2.5. G-Code Output

The path planning between subparts in this article is planned as follows: When a sub-part is printed out, rotate the receiving platform until the current base cross-section is parallel to the horizontal plane, so the next adjacent sub-part can be built perpendicular to the horizontal plane. According to the conclusions in reference [25], a printed model would have best surface quality when built perpendicular to the horizontal plane. Every time a subpart is printed out, the receiving platform needs to go back to the original space location by rotating the same angle in the opposite direction. Then, the next sub-part starts to print.

The rotation angle is determined by the normal vector of the base cross-section ${\;S}_{i}.\;$ Assuming the current normal vector is $n_{i}\left(\;x_{i},\;y_{i},\;z_{i}\right)$, the calculation process of the angle is the same that of Equations (7) and (8) in Section 2.2.4.

Due to the rotation of the receiving platform, the space location of the next adjacent sub-part changes, and this needs to be updated by the matrix transformation (which is same as the matrix of Equation (9) in Section 2.2.4). The subpart can be sliced using the method in Section 2.2.4 after the spatial location of it is updated.

During the process of subpart slicing, traverse the slice parameter list of this part and carry out the above-mentioned dynamic slicing operation until the slice parameter list is traversed, and then a complete slice layer list is finally obtained. The slice layer list is iterated to obtain the point coordinates of each print line segment $p_{0}$ and $p_{1}$. Note that the output of print line segment in the computer is composed of the parameters of the starting point $p_{0}$ and the ending point $p_{1}$.

As printing G-code of each subpart is obtained by dynamic slicing method, each subpart is associated with the base cross-section; therefore the integral printing G-code is achieved by combining each G-code of each subpart based on the relationship between subparts.

### 2.3. Experimental System

(1) Hardware platform

**Figure 10:**
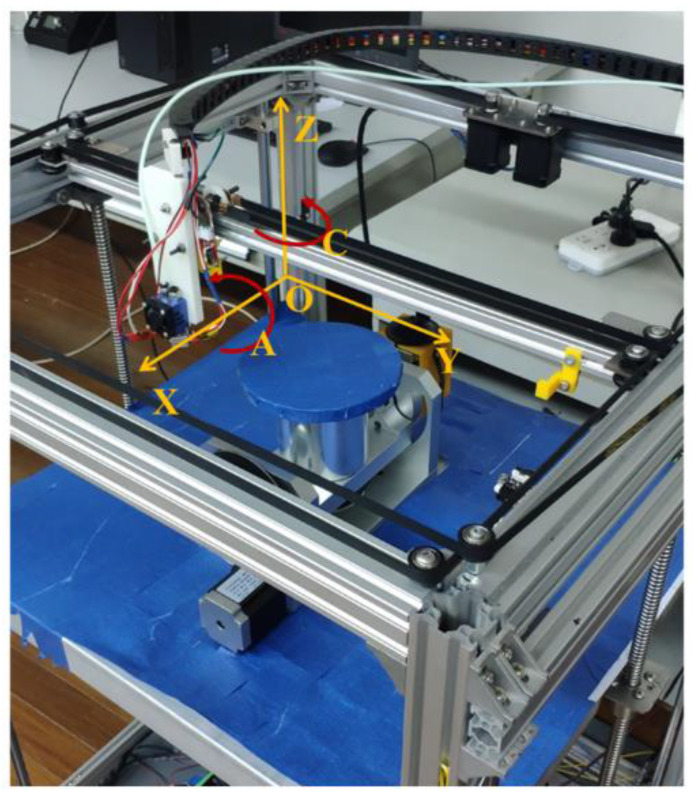
Five-axis printing platform.

A five-axis printer was developed, as shown in Figure 10. The device was improved based on the X–Y transmission mechanism and equipped with a receiving platform. In order to ensure higher mechanical transmission stability and excellent mechanical properties, the main printing platforms and guide rail are made of metal materials. The moving ranges of X, Y, and Z are 500, 500, and 700 mm, respectively. The moving angles of axes *A* and *C* are 180 and 360 degrees, respectively

(2) Software system

**Figure 11:**
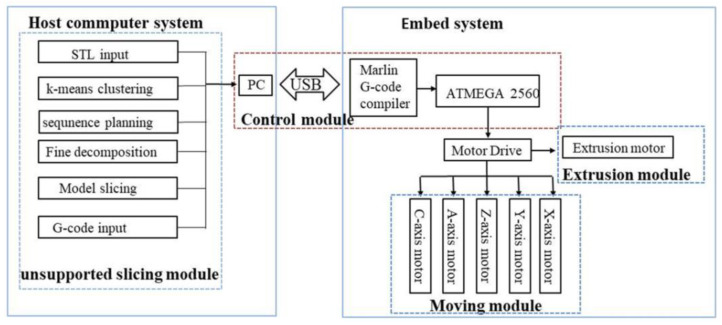
Non-directional unsupported 3D printing system.

As shown in Figure 11, the non-directional unsupported 3D printing system is mainly composed of a host computer and an embed system. The main function of the host computer is to generate the G code of five-axis printing, including STL model input, model coarse partition, sequence planning, model slicing, and G code generation. The host computer uses the Repetier-Host software to load the G-code obtained from the Python-unsupported slicing module into the software, and it uses output G-code to communicate with the motion control module of our embed system. The embed system uses open-source Arduino software to develop and realize the control of the five-axis printer.

## 3. Results

### 3.1. Printing Sample Display

**Figure 12:**
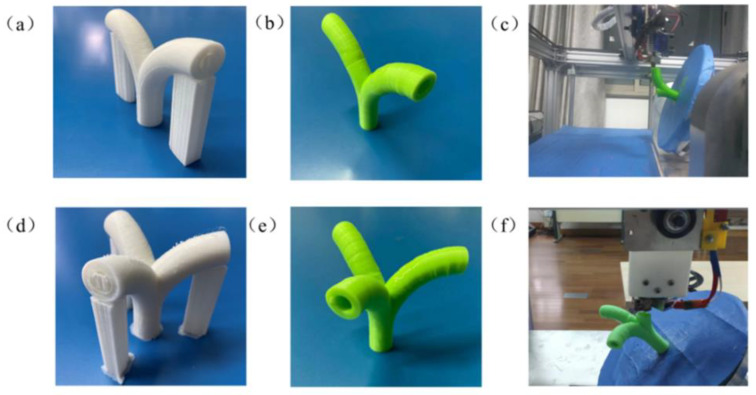
Multi-branched structure model printing: (**a**) two-branched pipe printed by conventional 3D printing, (**b**) two-branched pipe printed by non-directional unsupported printing, (**c**) the non-directional unsupported printing process of the two-branched pipe, (**d**) three-branched pipe printed by conventional 3D printing, (**e**) three-branched pipe printed by non-directional unsupported printing, and (**f**) the non-directional unsupported printing process of the three-branched pipe.

As shown in Figure 12, the printing experiments used degradable natural thermoplastic polylactic acid (PLA) as printing materials. Different colors were used to display two printing methods. The printing results of the two-branched pipe in traditional three-axis printing are shown in Figure 12a, while the non-directional unsupported printing results of the model are shown in Figure 12b. Additionally, the two methods were tested on through a pipe with three branches. Figure 12d shows the printing result of traditional three-axis printing method, while Figure 12e shows the five-axis printing method. Figure 12c,f show the process of printing models in the five-axis system.

During a conventional 3D printing process, the forming direction of the model is always stacked layer by layer from the bottom to the top along the *Z*-axis direction, so the support structure is inescapable while the model has multiple overhanging structures (e.g., Figure 12a,d). The overhanging branch part requires a certain amount of support materials to be printed out, and the inner holes are filled with the supporting materials that are difficult to peel off. In addition, there are some obvious burrs on the surface of the model. As shown in Figure 12b,e, these models with overhanging branches were all implemented with the non-directional unsupported 3D printing method. These models did not contain supporting materials in the inner hole and the external outline. No post-processing was required to remove the support structure.

### 3.2. Printing Parameters Analysis

The non-directional unsupported 3D printing process parameters are shown in Table 1. According to Table 1, the non-directional unsupported 3D process can avoid supporting materials to a greater extent than conventional 3D printing. The non-directional unsupported 3D printing requires a much lower amount of additional materials to generate objects than conventional 3D printing, the printing process is faster, and the printing efficiency is significantly improved. Compared to the conventional 3D printing method, the non-directional unsupported 3D printing can save about 18.72–20.60% of materials and about 20.60–23.33% of time.

However, there are still some limitations. First, when printing one of the subparts of the multi-branched model, because of the accuracy of the rotary motor axis of the receiving platform and the rigidity of the hardware device, this will lead to errors in nozzle positioning, resulting in the misalignment of the printing model. The machine should be equipped with error feedback and correction system. Second, as the model is divided into blocks, when the first subpart is printed, it will be used as the reference support of other subparts. Due to the long time cooling of the materials, the bonding degree between the subparts is poor; this will be a task for future research.

## 4. Conclusions

In this paper, a non-directional unsupported 3D printing method was proposed to realize the unsupported 3D printing of multi-branched models. By implementing model decomposition, the complex multi-branched models turned to different simple parts, so the dynamic slice method was adopted to each part to obtain the printing G-code. A multi-branched overhang model can be printed without support structure added. As a result, some multi-branched models were tested using the non-directional unsupported printing method on the five-axis printing platform, achieving the desired results. A comparison of printing materials and time showed that the non-directional unsupported 3D printing could save 18.72–20.60% of materials and 20.60–23.33% of time compared to conventional 3D printing. Therefore, the printing materials consumption and efficiency of a multi-branched model using the proposed method were significantly improved compared to normal 3D printing. Besides, the non-directional unsupported 3D printing method never required post-processing to remove the support structure.

However, toward the objective of completely eliminating the support, there are some obvious limitations for the proposed printing method on the five-axis printing platform. First, the input geometry must have a clearly identified single flat base as the root, since it plays the role of supporting the adjacent subparts. Second, the non-directional unsupported 3D printing method aims to print the tree-like branched structure geometry, which means explicit and meaningful segmentation can be obtained using the K-means clustering algorithm, but the models that do not have clear block geometry feature are very likely to fail during our decomposition process. There is a chance that models that incorporate these two features may never be support-free.

## Figures and Tables

**Table 1:** Comparison results of slicing effect.

Model Name	Slice Thickness (mm)	Consumed Materials (mm)		Decrement (%)	Printing Time (s)		Decrement (%)
		Cura	5-Axis		Cura	5-Axis	
two-branched pipe	0.1	10236	8216	19.73	15697	12234	22.06
	0.2	10346	8216	20.59	7988	6217	22.17
	0.3	10347	8216	20.60	5417	4153	23.33
three-branched pipe	0.1	11560	9396	18.72	18322	14572	20.47
	0.2	11565	9400	18.72	9152	7236	20.93
	0.3	11570	9403	18.73	6113	4854	20.60
